# 1309. Fungal Prosthetic Joint Infection - Case Series and Review of the Literature

**DOI:** 10.1093/ofid/ofad500.1148

**Published:** 2023-11-27

**Authors:** Victoria Starnes, Joan Duggan, Caitlyn M Hollingshead

**Affiliations:** University of Toledo College of Medicine and Life Sciences, Maumee, Ohio; University of Toledo Medical Center, Toledo, Ohio; University of Toledo College of Medicine and Life Sciences, Maumee, Ohio

## Abstract

**Background:**

The most effective treatment for fungal prosthetic joint infections remains unclear. Most cases are treated with two-stage revisions combined with systemic antifungal medications. To date, the largest studies of total hip arthroplasty and total knee arthroplasty fungal infections have included 37 and 45 patients, respectively.

**Methods:**

A retrospective record review of patients admitted in two health systems between January 1, 2007 and December 31, 2018 with prosthetic joints and a deep culture of the joint positive for fungal organisms was performed as well as a review of the literature. A Pubmed and Embase search of the English-language literature from Jan 1, 1980 to Jan 1, 2023 was performed with review of the pertinent references for cases meeting the following case definition: individual with prosthetic joint and positive deep tissue culture for fungus.

**Results:**

159 patients fit criteria. 73 patients had knee replacements, 62 patients had hip replacements, and 5 had other joint involvement. 52% were female. 137 patients had yeast involvement, with *Candida* species being predominant, while 11 had mold and 11 with dimorphic infections. 55 patients were treated with two-stage revisions, 44 received one-stage revisions, 32 received debridement only or Girdlestone procedure, and 1 required amputation. 141 reported details on antifungal therapy. After performing multivariate analysis, polyene treatment was found to be associated with higher rate of recovery, p=0.042. However, there was a trend towards recurrence requiring surgical intervention in patients treated with polyenes, p=0.067. 22.6% had a poor outcome, including recurrence, amputation, or death.

**Conclusion:**

Surprisingly, polyenes did not underperform when compared to other antifungal therapy. Prospective research assessing optimal surgical treatment modality and antifungal therapy is needed as this infection is associated with high morbidity and mortality.

**Disclosures:**

**All Authors**: No reported disclosures

